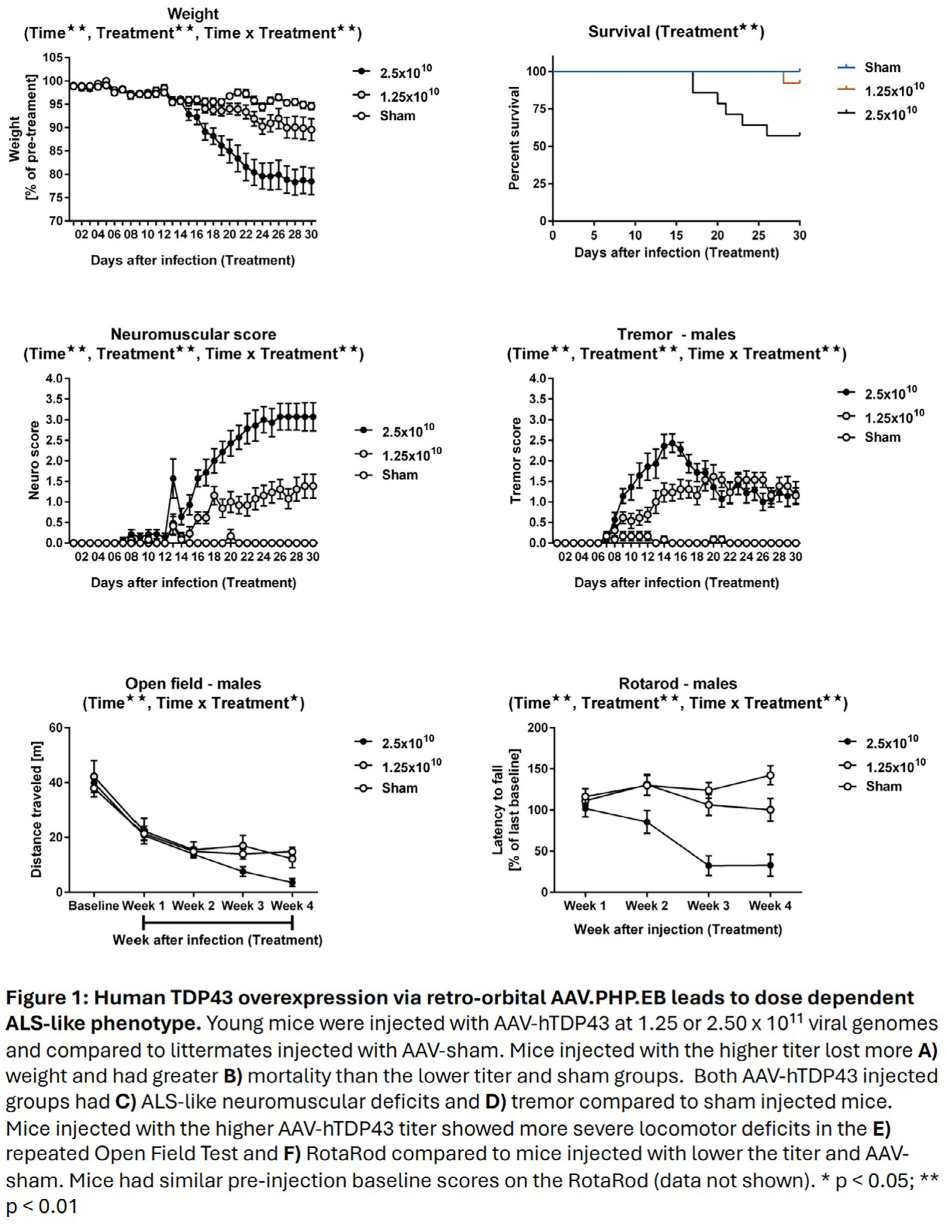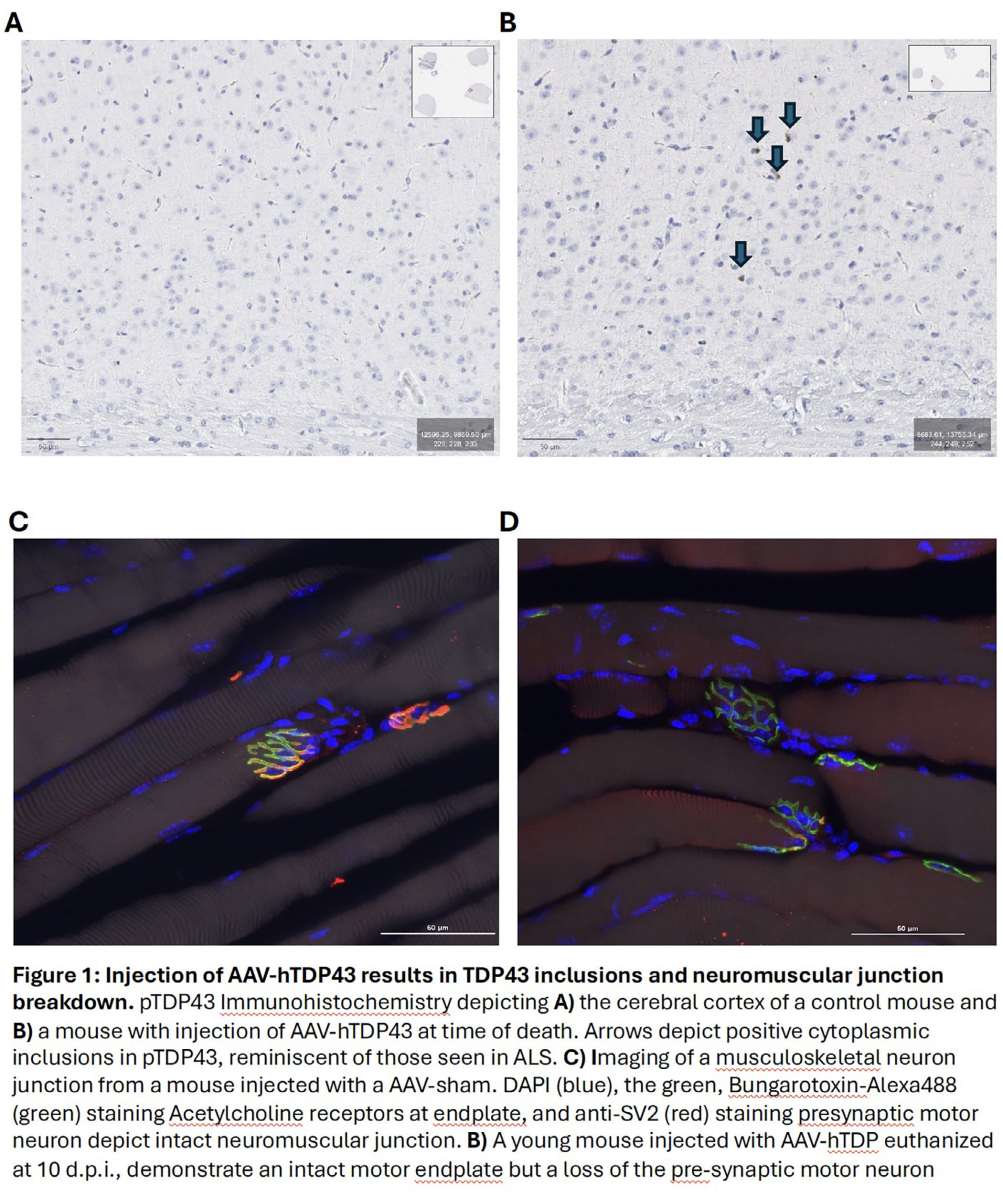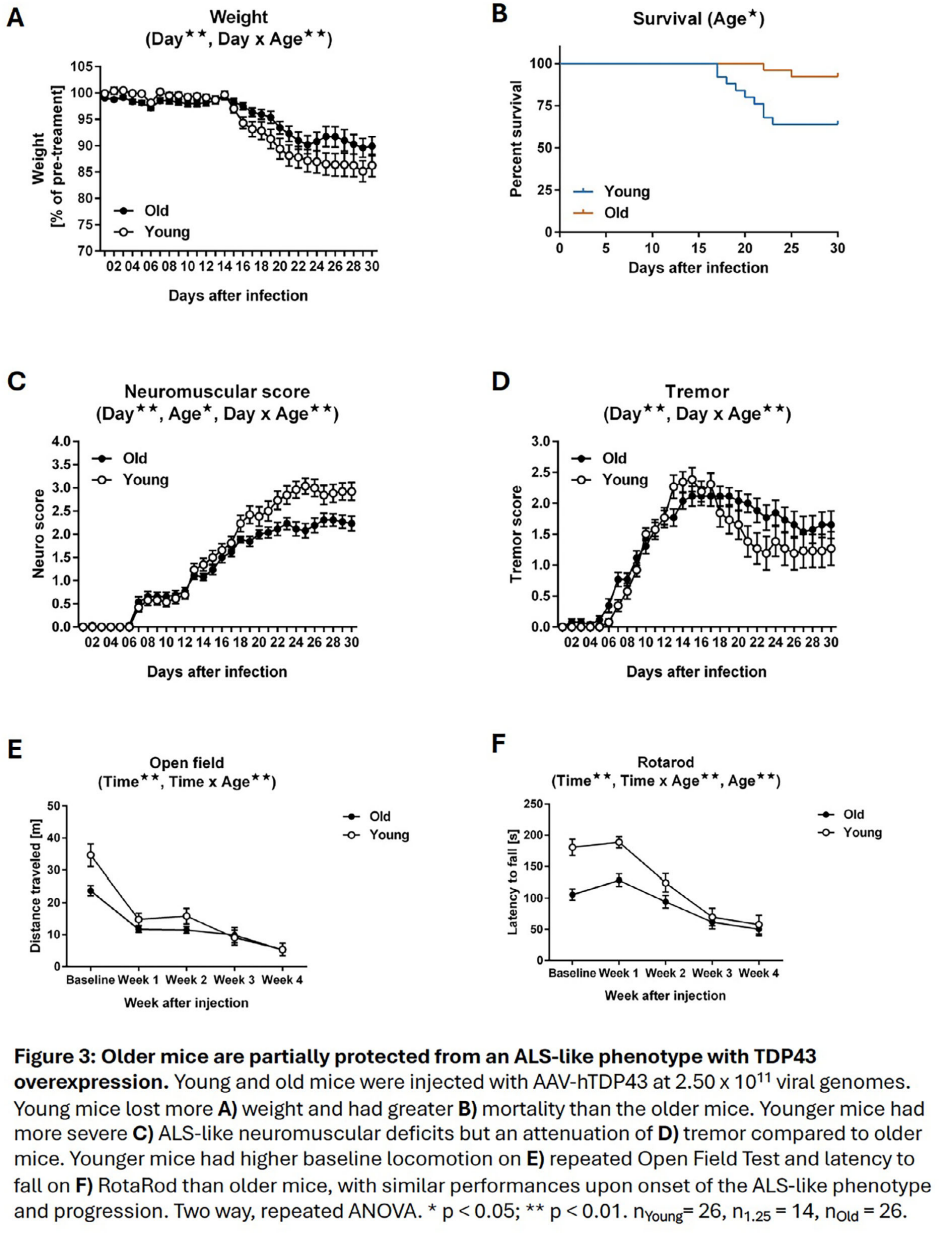# Novel Mouse Model of TDP43‐opathy Demonstrates Paradoxical Effects of Aging on Neurodegenerative Phenotypes

**DOI:** 10.1002/alz70855_105723

**Published:** 2025-12-24

**Authors:** Daniel W Fisher, Caitlyn Schaffer, Christian Battaglia, NhiVan Tran, Nzinga Hendricks, Alec ST Smith, Martin Darvas

**Affiliations:** ^1^ University of Washington, Seattle, WA, USA

## Abstract

**Background:**

Aging is the greatest risk factor for neurodegenerative diseases like ALS, but studying how specific aging processes alter pathogenicity has been challenging. Though neurodegeneration is often linked with pathological protein aggregation, it is unclear whether aging simply allows the time necessary for proteins to aggregate into pathological forms or if concomitant aging‐related processes further facilitate aggregation or resultant neurodegeneration.

**Methods:**

We developed a TDP43‐opathy/ALS mouse model by retro‐orbital injection of a viral vector for neuron‐restrictive human TDP43 overexpression, AAV‐Syn‐hTDP43‐Php.Eb (hTDP43). Young (5‐6mo) and old (21‐23mo), male and female mice were administered AAV‐hTDP or AAV‐Sham with daily measurements of weight, neuromuscular score (NMS; 0‐4), and tremor severity (0‐4). Weekly open field, y‐maze, and rotarod tests were administered. Mice were euthanized at NMS = 4 or at 30 days post‐injection (dpi) and the CNS or neuromuscular junction was fixed in 10% formalin, fixed with, 4% PFA, or frozen on dry ice. Immunohistochemistry was performed with commercial antibodies. qPCR with primers specific to the viral vector were performed on spinal cord DNA.

**Results:**

Young mice injected with AAV‐hTDP43 developed a tremor at about 7 dpi that progressed in severity, with worsening neuromuscular impairment, weight loss, and death. The speed and severity of motor deficits, weight loss, and time to mortality were titratable by dose. Sparse but clear pTDP43 inclusions were noted in the spinal cord and layer 5 cortical cells. Surprisingly, when old and young mice were injected with a viral titer projected to cause 50% mortality, older mice demonstrated significantly less weight loss, lower NMS, and less mortality than young mice. Similar viral titers were detected in the spinal cords of old and young mice, excluding the likelihood of age‐related changes in viral infectivity.

**Conclusions:**

We developed a novel ALS‐like mouse model that allows for tight temporal and dosage control of resultant pathology. This tool can enhance efficiency in experiments studying complex genetics or aging in TDP43‐opathies. Surprisingly, when young and old mice were challenged with acute hTDP43 overexpression, aging played a protective role in the subsequent ALS‐like phenotype. Subsequent work will explore the mechanisms behind this age‐related resilience.